# An App about Healthy Habits as an Educational Resource during the Pandemic

**DOI:** 10.3390/healthcare10010148

**Published:** 2022-01-13

**Authors:** María de los Ángeles Merino-Godoy, Emilia Moreno-Sánchez, Francisco-Javier Gago-Valiente, Emília Isabel Costa, Jesús Sáez-Padilla

**Affiliations:** 1 Nursing Department, Faculty of Nursing, University of Huelva, 21007 Huelva, Spain; angeles.merino@denf.uhu.es; 2 Department of Pedagogy, Faculty of Education, Psychology and Sports Sciences, University of Huelva, 21071 Huelva, Spain; 3 Health Department of IES Cuenca Minera, Minas de Riotinto (Huelva), Consejería de Educación y Deporte, Junta de Andalucía, 21007 Huelva, Spain; francisco.gago@dstso.uhu.es; 4 Nursing Department, Health School, University of Algarve, 8000 Faro, Portugal; eicosta@ualg.pt; 5 Health Sciences Research Unit: Nursing, Nursing School of Coimbra, 3000 Coimbra, Portugal; 6 Integrated Didactics Department, Faculty of Education, Psychology and Sports Sciences, University of Huelva, 21071 Huelva, Spain; jesus.saez@dempc.uhu.es

**Keywords:** innovation, healthy habits, health education, mobile application, educational resources

## Abstract

Educational institutions and their agents play a fundamental role in improving people’s health literacy and quality of life. We intend here to describe and justify an educational resource embodied in an application for mobile devices developed through a subsidized project by the Ministry of Health (Government of Andalusia); the purpose of this app is to educate young people in healthy habits. The application was designed to be easily used in both smartphones and tablets with the aim of achieving good physical, psychological and social health. The project comprises several phases and the results we have so far show that, from an early age, health institutions and educational settings must work in partnership, increasing health literacy levels. This cooperative work combined with the use of this innovative approach presents an important potential for change in the lifestyles of younger generations. This type of intervention took on a special role in the pandemic context, allowing for the maintenance of the educational stimulus in a safe context.

## 1. Introduction

The contents of health education (HE) are usually part of the so-called cross-sectional axes of the curriculum, such as education for the environment, road safety and equality in the initial and continuous teacher training plans, although these aspects encompass important social and early childhood health problems.

According to the World Health Organization (WHO), a current concern is obesity, which is the “epidemic of the 21st century”, and it involves genetic risk factors, as well as other factors that can be modified and controlled.

The causes of the overweight epidemic include the profound changes in our lifestyles, which constitute the multiple factors that contribute to the so-called “obesogenic environment” [1]. Political and educational actions must be aimed at mitigating anomalies in these determinants. We are facing a problem that may not have been properly valued to date, given the hazard that it poses to people’s quality of life. It has been demonstrated that child obesity is a socio-sanitary problem influenced by non-modifiable risk factors (e.g., genetic factors) [2]; however, it is also influenced by other factors on which we can act, such as unhealthy eating habits and physical inactivity, although the socio-economic status of the family also influences child obesity [3], as it has an impact on the family and individual lifestyle.

One of the characteristics of the sudden changes of current society is the advance in new technologies, which have caused important changes in the habits and lifestyles of people, impacting health. “The new social models facilitate informal learning environments in which users acquire competences intentionally and/or unwittingly, without a pre-established curriculum and in an essentially extracurricular context, based on technological-digital platforms and stimuli” [4] (p. 288).

Within the healthcare policies of the WHO and of the European Union, the Spanish Ministry of Healthcare, through the Spanish Agency of Food and Nutrition Safety (AESAN), implemented in the year 2005 the Strategy for Nutrition, Physical Activity and Prevention of Obesity (NAOS). These actions have mobilized public administrations, food sector and other public and private agents, scientific societies, consumer organizations, etc., to sensitize and raise awareness in the population about the problem posed by overweight and obesity, which is considered by the WHO as an epidemic of the 21st century.

An initiative in this regard is the ALADINO study (Eating, Physical Activity, Child Development and Obesity), which was developed with the aim of estimating the prevalence of overweight and obesity in the population of children aged 6–9 years. The design of the ALADINO study is adjusted to the protocol of the COSI initiative (WHO European Childhood Obesity Surveillance Initiative) of January 2008 [5].

In our context, the Government of Andalusia [6] approved the project entitled “Bill for the Promotion of a Healthy Lifestyle and Balanced Diet”. The text foresees the realization of the Andalusian plan for the promotion of physical activity and healthy diet with measures that will be expanded to educational centers. Article 42 of this Law gathers the actions of new technologies and physical activity, pointing out that the relevant councils in matters of health and innovation will work jointly to promote the development of games that use new technologies and increase physical activity in different profiles of users in an inclusive manner. This argumentation justifies the large number of studies showing that beyond the family environment, mobile devices, especially tablets and smartphones, have replaced computers in schools [7,8,9].

Specifically in the educational scope, the Organic Law for the improvement of the quality of education [10] proposes that educational administrations will adopt measures to ensure that physical activity and a balanced diet become part of children’s and young people’s behavior. As is proposed by other educational actions, teachers will have to design, coordinate, and supervise the measures adopted in the centers (the fourth additional provision).

The reality is that this regulation is not always assumed, in some cases due to a lack of training and in other cases due to a lack of sensitivity in the approach on health education in the curricula of early childhood and primary education. However, its incorporation in the Spanish school system is undoubtedly suitable and necessary, especially for the promotion and development of healthy lifestyles in childhood and adolescence [11].

In this line of work, we propose an innovative educational project that aims to promote, among other actions, a culture of prevention of childhood obesity, which is one of the risk factors for numerous diseases and psychological problems in a child-youth population aged 8–16 years. Through this app, healthy eating habits and physical activity are worked out, and other aspects related to health are addressed, such as interpersonal relationships, interaction with new technologies and social networks and the risk of addiction.

In this way, the main objective of this project is to promote knowledge about healthy eating habits and the practice of physical activities, to improve the quality of life of students of primary and secondary education. These actions will also encourage responsible food consumption by the population and, thus, the sustainable economy of the regions that participate in the project. Therefore, a cross-border innovation project was designed which consists of four phases, within the framework of the INTERREG VA Spain-Portugal Programme (POCTEP) of 2014–2020 [12].

Andalusia (Spain), Algarve and Alentejo (Portugal) are regions with demographic weakness, low employability, and poor innovation. In general, these are recognized regions with special needs within the POCTEP. This project benefits nearby geographic areas with common characteristics (climatic, economic, lifestyle, etc.) and needs. In fact, regarding this matter, it is important to highlight that Andalusia is the autonomous community with the highest rate of obesity, with an increase of 7.4% in the last 25 years, and two out of 10 children aged 10–17 years are overweight or obese according to data from the Spanish Institute of Statistics in 2013. These data show a rate of 7.93% obesity in this age range in 2017 [13].

The project was designed to promote cooperation throughout the border between the two countries to improve the quality of life of the population. The study space covers areas of Andalusia (Huelva, Ayamonte and Aljaraque), Algarve (Faro, Vila Real de Santo Antonio) and Baixo Alentejo (Beja). These actions allow the advancement of health and quality of life in these regions which share geographic, demographic, and cultural characteristics, and they have resources mainly oriented toward the primary sector according to the European Commission. Moreover, it is intended to: (1) improve the institutional capacity and efficiency of the public administration through cross-border cooperation; (2) design actions for a healthy diet and exercise that involve the school community of the educational centers (families, students, teachers and members of the Parents Association (AMPA)); (3) foster the participation of citizens in the improvement of their life habits and communication with the public administration, promoting the proper use of ICTs; (4) increase the economy of the local natural resources through the consumption of seasonal products of the region; (5) favor the innovation of the agro-food industry; and (6) promote intelligent mobility.

These actions are aimed at sensitizing and training the members of the educational community, promoting practices that will improve the quality of life of the child-youth population from the stage of primary education, as well as collaborating in the didactic use of a mobile application (app) that is simple, entertaining, easy to use and scientifically rigorous. This resource will allow experiences to be socialized, and it is designed to inform children and young people about habits that improve physical, psychological, and social health in school hours and in other moments and contexts.

With this innovative project, we intended to achieve a change of paradigm within a salutogenic model focused on observing and tackling the origins of health and well-being, as well as to create a health culture that guarantees the participation of the entire educational community.

In the development of this project, we started from the evidence that indicates that the risk factors for childhood obesity are associated with sociodemographic characteristics (gender, educational level of the family and nutritional status) and health-related lifestyle habits (physical activity and sport, for example) that develop at this stage of life. We therefore believe that the development of an app and its implementation in educational contexts will improve the initial parameters and, thus, reduce childhood obesity values, especially in this pandemic period, during which values have increased exponentially.

## 2. Materials and Methods

This proposal is presented within a cross-border (Spain–Portugal) project, and it uses new information technologies as allies of healthy behavior education in children and young people, as these are stages in which the health habits and behaviors are consolidated. There is an important lack of knowledge in this population about how to incorporate healthy habits into their daily lives, and the challenge will be to provide them with the possibility of acquiring this knowledge in a simple, enticing, and enjoyable way. This coincides with the rise of the use of mobile devices (mainly smartphones and tablets) and with the scientific evidence of the impact of healthy apps on public health. In the current context, it is important to emphasize that different studies have reported that apps are helping in the struggle against COVID-19 [14,15,16].

As previously mentioned, this project is based on the development of a digital application (for digital devices such as mobile phones or tablets) aimed to educate young people in healthy habits. This proposal (project code PIN-0445/2016) has been funded with 27,000€ by the Health Council (Resolution of 20 December 2016, of the General Secretariat of Research, Development, and Innovation in Health; in this call, grants were given to fund biomedical research, development, and innovation in life sciences in Andalusia for the year 2016).

**Figure 1:**
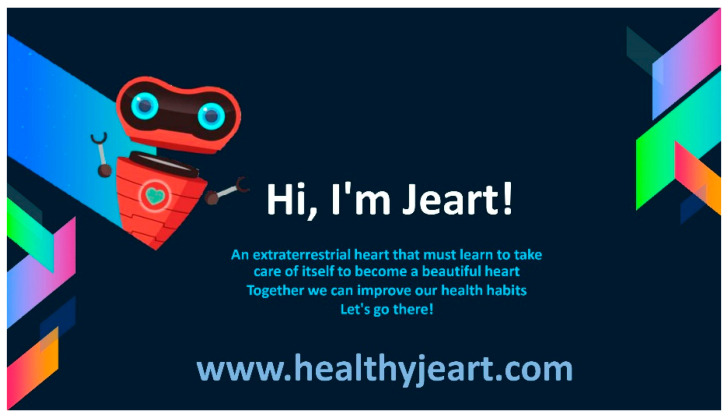
Presentation of Healthy Jeart.

Our goal is to promote the use of this simple, entertaining, easy-to-use, and scientifically rigorous mobile application called Healthy Jeart (Figure 1) as a health education resource in educational centers of the regions that participate in this activity. This application is currently being evaluated by the Health and Education Council of the Government of Andalusia to be include as a tool within the program of healthy habits taught in school centers, entitled “Growing in health” in the case of primary education and “Young Shape” in the case of secondary education. To this end, the Andalusian Agency for Health Accreditation (ACSA) processed a series of actions, obtaining the distinction of “healthy App” (Appendix A). The purpose of Heathy Jeart (Figure 1) is to educate the child-youth population aged 8–16 years in healthy habits.

This application was created to facilitate the acquisition of healthy behaviors. In this app, through a game and a forum of shared ideas, the users can learn positive attitudes and adopt them in their daily routine. All this is reinforced with healthy tips that can be read throughout the use of the application. It was designed as a tool for health promotion and the prevention of unhealthy behaviors, fostering empowerment in the self-care of health through healthy habits. The priority intervention area is the child-youth population, not only in the healthcare scope, but also at the educational and community levels.

For the realization of this project, several inter-linked applications were developed: (1) CMS: a web application that users can access as administrators, from which they can manage the contents of the applications. This back-end is in Php-MySQL language. (2) App iOS and Android: a native application compatible with iOS and Android devices, through which the users can check the contents and participate in the activities of Healthy Jeart. These apps are in Objective-C and Java languages, respectively. (3) Unity Game: an action game developed in Unity, accessible from the native apps. This game was developed in Unity, in C# language.

This pioneering project is structured in four phases, with the aim of attaining a series of objectives in each of them. In a first phase, an initial study/diagnosis was conducted to identify the health habits and the causes that influence child obesity in the scholar population. In addition to these aspects, there are currently other important problems for all ages, such as the effects of the health alarm generated by the Coronavirus pandemic (COVID-19), which worsens their health conditions and well-being [17]. This social scenario, which has taken place all around the world, poses new challenges and uses of the resources that have been employed in daily living, health, and education, as priority factors. Several studies show that close contact is one of the variables that influence the impact of SARS-CoV-2 [18,19]. Hence, during the pandemic, the classical contexts where educational processes are developed were identified as risk environments, and it was necessary to find new ways to maintain teaching, such as resorting to new technologies.

In the context of this project, all this problematic is analyzed within an interpretative paradigm, since we do not only consider the phenomena of interest, but also their relationship with the context in which they take place. The obtained results, in the previous phase, are used to design, in the second phase, an intervention adapted to the findings. This is a work of innovation in the establishment of better practices in the educational processes about nutrition and physical activity, among other aspects, increasing the means of communication, such as the use of an app about healthy habits and forums as didactic resources, proposing changes in the curricular contents in primary and secondary education.

**Figure 2:**
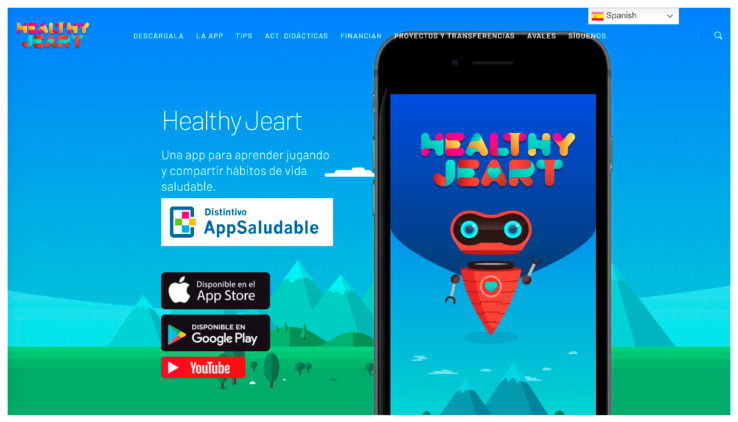
Mobile application available for Android and iOS. An app for learning by playing and sharing healthy lifestyle habits.

The third phase includes the implementation of the actions involved in the development of the intervention. At this point, we established the following action lines: (a) community educational training (families and students, through face-to-face or online discussion groups, courses and forums); (b) workshops (preparation of recipes in school activities, fostering the consumption of seasonal products); (c) training for the didactic use of the Healthy Jeart app; and (d) creation of a website to communicate and disseminate the activities and results of the project and for the electronic participation of the educational community (Figure 2) (www.healthyjeart.com). We emphasize that the website “Healthy Jeart” obtained the ACSA awarded seal of accreditation at the advanced level for web pages (Appendix B).

In the fourth phase, where this project is currently positioned, we will evaluate the impact of the workshops and app utilization, and we will prepare the technical report on the use of the Healthy Jeart app and website, as well as pedagogical guidelines to optimize the use of this resource in health promotion interventions.

## 3. Results

There is a considerable lack of knowledge in this population about how to incorporate healthy habits in their lives, and the challenge is to give them the possibility to attain such knowledge easily and quickly. This coincides with the rise of the use of mobile devices (mainly smartphones and tablets) and with the scientific evidence that shows the impact of healthy apps on public health.

There is an increasing number of apps that tackle some aspect of health, although most of them lack scientific rigor, with very few of these apps being specifically aimed at children and young people. The most popular and successful healthy apps are Endomondo, the Nike+ Running App, Viquiz, Esporti Revolutio and El Círculo de la Salud [20]. In our case, after a thorough effort, we designed a mobile app that meets all the scientific criteria. The aim of this study is to respond to the needs of implementing it in the educational centers of the cross-border environment based on the characteristics of the area and the permanent collaboration that has been maintained between Algarve, Alentejo and Huelva in activities of health education.

**Figure 3:**
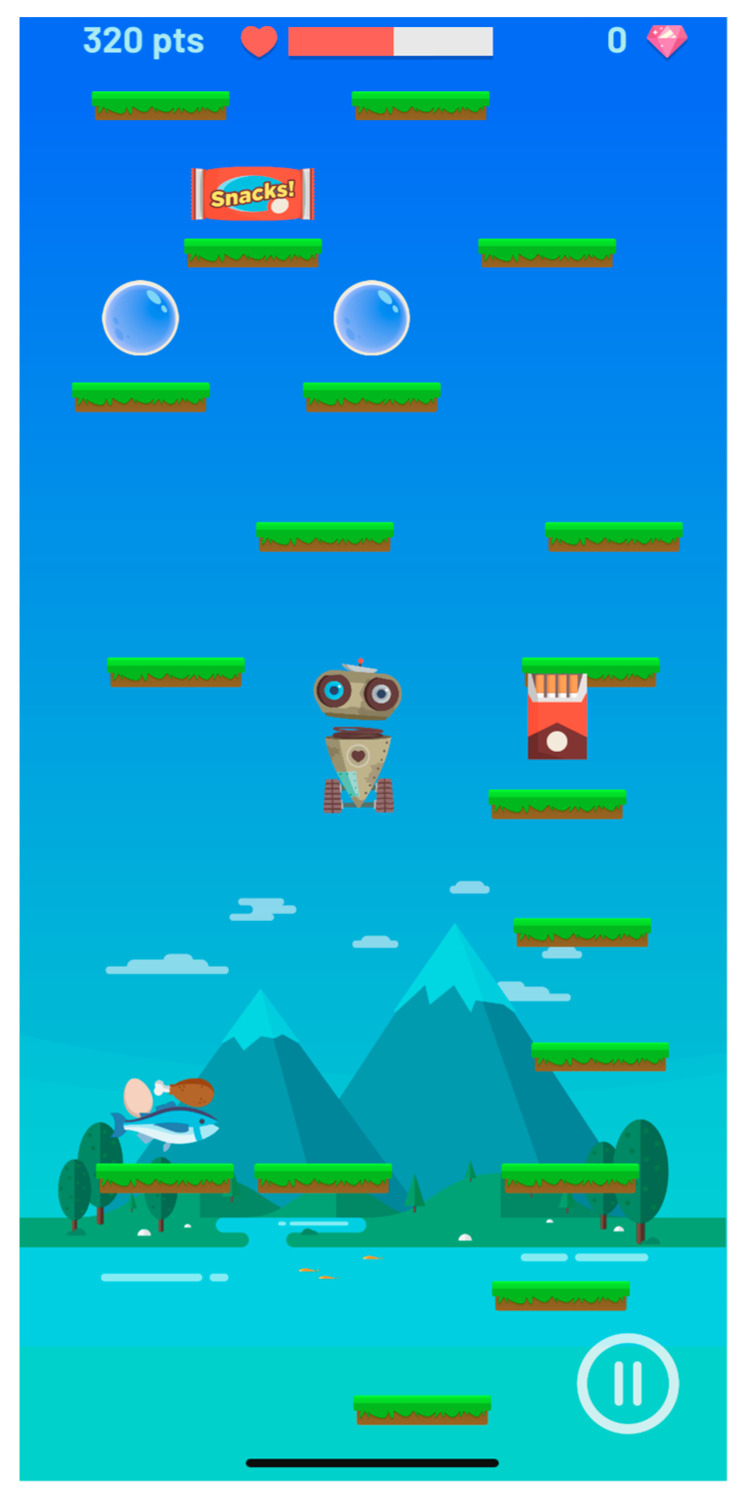
A game to work on healthy habits playfully.

The app consists of four main sections; thus, their joint work favors the learning of healthy habits:

a. Game. With gamification, the user learns healthy habits while having fun and playfully helping Jeart in a frenetic game as it goes up and gathers healthy elements that prolong its life, avoiding other, harmful elements. On its way, it will find rest elements and protection shields. The game allows the user to stop whenever he/she wants, and, upon failure, the app asks the user a question related to health, which will allow the user to resume the game where he/she left it, if the answer is correct (Figure 3).

The user can propose ideas that promote healthy habits, and the administrators will score them. The former will receive a notification of this score on their devices. They will be able to use this score in the game to improve the health of other participants.

b. Health tips and didactic resources. This section fosters the modification of habits through small knowledge “pills”. Thus, ordered in seven knowledge areas (Physical Activity, Eating, Physical Well-being, Psychological Well-being, Toxic Substances and Addictions, Sexual-Affective Dimension and New Technologies), the user can check advice related to healthy habits represented in brief chips or tips. Moreover, the user will be able to share the tips in his/her social network with a single click. This will provide direct and clear information with brief and simple messages backed by experts and adapted to the language of young people. Furthermore, in these seven sections, teachers can directly access didactic activities created by experts, which, with two clicks, they can share and perform in the classroom, thereby experiencing the capacity to become empowered and responsible of their own health.

**Figure 4:**
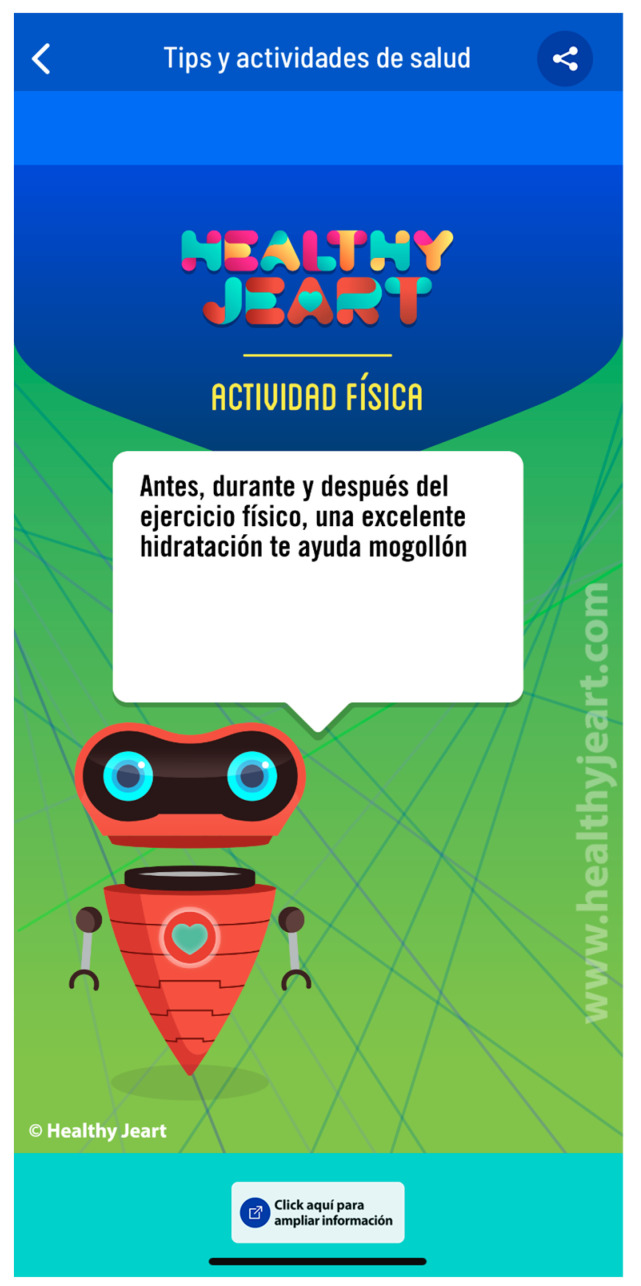
Healthy tips and didactic resources. “Before, during and after physical exercise, an excellent hydration helps you a lot”.

The administrators can create health challenges that the educational centers can join. These challenges have a start and finish date. The participants of each center will receive points if they complete them (Figure 4).

c. Forum of ideas. The users who wish to register can easily send, from the app, a brief idea of how to improve or implement a healthy habit. This idea is revised and scored by the administrators, who grant stars that can be exchanged for points in the game to modify the appearance and resistance of Jeart. These ideas, as well as the user’s nickname, are gathered and presented in the app, thus making them visible to everyone, fostering the participation of young people.

Whoever uses the application can play a game that rewards the acquisition of healthy items. At the end of the game, the player can use the points earned to make the appearance of Jeart evolve into an increasingly healthy image. Any user of the app can create healthy ideas and write them in the app which will then be evaluated; based on such evaluation, they will earn gems for the game.

d. Challenges. From the app, a monthly healthy challenge is established for the educational centers, which must be completed within approximately 21 days (time estimated to modify habits). Next, the teacher must enter the app and describe his/her experience. The different participations are evaluated, and the winning center is given a certificate (issued directly by the app), in addition to an award that recognizes its work for the modification of healthy habits in the classroom.

The app works on six areas: eating, physical activity, physical well-being, psychological well-being, toxic substances and addictions, and the sexual-affective dimension. In this sense, healthy eating is multi-factorial, thus it is influenced by the lifestyle and habits of the population.

## 4. Discussion and Conclusions

From the World Health Organization, as was stated by Halfdan Mahler [21], primary care is one of the pillars for the improvement of health worldwide. Health professionals, despite the current difficulties arising from the COVID-19 pandemic, must engage in new roles and adopt new perspectives, allowing them to respond to contemporary challenges, namely those related to globalization and the growing technification of the world.

One of the most important strategies will be collaborative work in partnership with educational agents. This aspect will allow the development of challenging initiatives, enhancing knowledge and interventions of each part, and providing the optimization of results, specifically, in this case, the expansion of health literacy levels. They must be motivated and prepared to design, practice, and adapt such initiatives, expand their boundaries, and develop propositions for the promotion of health, always considering the perspective of teamwork. Their main concern must be the development of ways to help individuals and communities to become more self-responsible and empowered in relation to their global health.

Thus, we live in a time in which health professionals must develop new skills that respond to the new health needs of individuals. This aspect is also relevant for the new generation of health professions’ educators; the training of these experts can no longer be limited to clinical contexts, health is built, promoted in different settings and with the collaboration of different actors.

It is, therefore, important to highlight that educational organizations constitute a privileged space for the prevention of health problems and the promotion of healthy life habits. Schools and families must actively engage in the prevention of health issues that are directly linked to unhealthy eating and physical inactivity; habits acquired from the first years of life. In the action plan of educational institutions, social and digital media occupy a place in the learning processes that is impossible to disregard.

Therefore, it is essential to continue to research, educate and work in partnership to empower teachers, families, and students in school contexts in order to promote healthy lifestyle habits. We intend to innovate interventions in health promotion in these contexts for these populations. We want to use digital language and digital play environments, so familiar to children and young people, as a means of communication and health education.

In this attempt to respond to the problem of child-youth obesity with new analytical perspectives, it is understood that working on the protection of health, in both research and teaching, is a long-term challenge for public health and education. Currently, it is necessary to thoroughly deepen the theoretical fundamentals of healthy habits acquisition and maintenance, especially the evidence on health assets and the evaluation of the interventions developed in terms of health promotion and disease prevention. The approach to public health centered on prevention and in a pathogenic model is changing and redirecting towards a positive public health. The traditional tendency focused on protecting and emphasizing the reduction of risk factors is being re-oriented toward the consideration that health actions are connected to the development of primary care and to the intervention that makes individuals, families and communities take greater control over their health and work to improve it [22].

This model revitalizes the promotion of health and increases the value of the idea of health assets, understood as any factor or resource that enhances the capacity of individuals, communities, and populations to maintain health and well-being [23]. Some empirical studies about health habits in school children have allowed identifying a list of health assets in students [24]. The assets identified are related to physical activity, body weight, self-perception of health, happiness and relationships with relatives and peers, among others. Along these lines, natural and outdoor environments gain special importance, due to the benefits and possibilities they offer for an integral, naturalistic, and healthy work [25].

Thus, after evaluating the habits around healthy eating and physical activity in primary and secondary education students, our project “Promotion of Health in the Cross-border environment of Algarve, Huelva and Alentejo” proposes the use of the mobile application, Healthy Jeart, in the educational centers to work on healthy habits. Subsequently, we will evaluate the effectiveness of this approach in the modification of habits. We consider that the Healthy Jeart app can be an important tool to act on risk factors of numerous diseases, such as child obesity, inadequate nutrition, and sedentary lifestyles, improving the quality of life of young people.

Recent data show that it is necessary to implement projects such as the one described here to favor the creation of a health culture that guarantees the participation of the entire educational community [26,27]. Given the lack of public policies that regulate the sale and distribution of foods and considering the power that the media and marketing tools have on children from a very young age, the school is a crucial place of influence to develop healthy habits.

We believe that this project will help to establish better practices in the educational settings related to nutrition, physical activity, and healthy lifestyle in general. Through training, we propose to encourage communication among the different actors involved in education. The findings will serve to highlight the need to introduce changes in study plans, since the child and youth populations must be able to make good choices in terms of health, being aware that a healthy lifestyle has an impact not only in the first years of life, but throughout the entire life cycle.

## Figures and Tables

**Figure A1:**
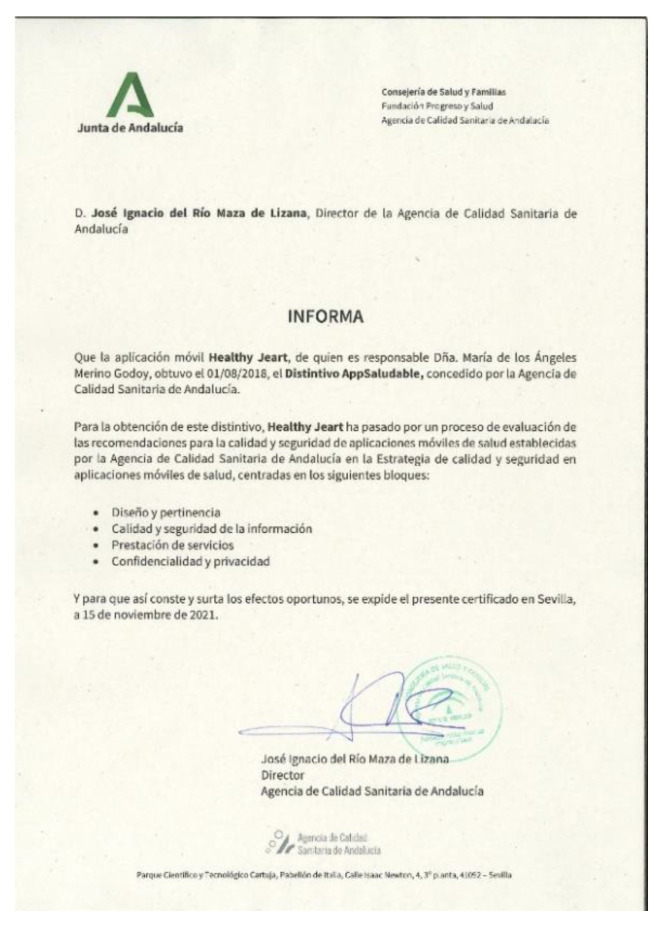
Distinction of “Healthy App”.

**Figure A2:**
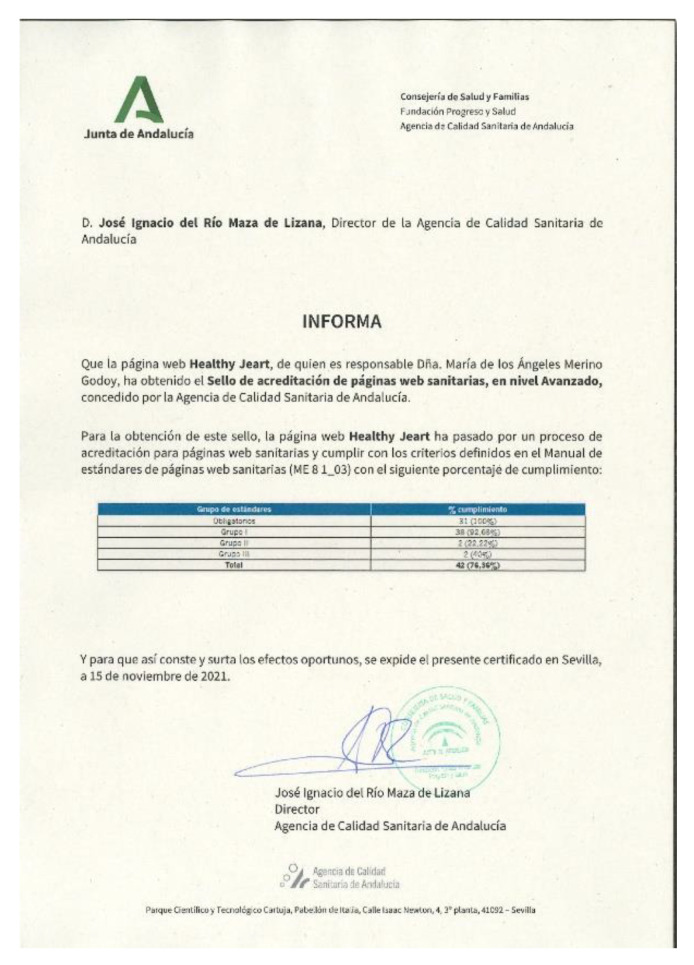
Advanced Level Accreditation for Web Pages.

## Data Availability

The data presented in this study are available on request from the corresponding author. The data are not publicly available due to privacy restrictions.
